# Reply: Randomised studies with translational end points are required to further elucidate the prognostic and predictive value of CA IX

**DOI:** 10.1038/sj.bjc.6603716

**Published:** 2007-03-27

**Authors:** S A Hussain, D W Rea, D H Palmer

**Affiliations:** 1 Cancer research UK Institute for Cancer studies, University Hospital Birmingham, Edgbaston, Birmingham B15 2TT, UK

**Sir**,

We have previously reported that CA IX (a marker of hypoxia) is an independent prognostic marker in early-stage breast cancer (Hussain *et al*, 2007).

A letter from Span *et al* in this issue questions whether CA IX is in fact a predictive factor for reduced benefit from adjuvant therapy rather than a prognostic factor. This is based on their own report of 253 patient series published previously (Span *et al*, 2003). Following the submission of our manuscript, a further publication has supported the conclusion that CA IX is a marker of poor prognosis in premenopausal breast cancer patients and it is an independent predictor of survival in patients with one to three lymph nodes (Brennan *et al*, 2006).

Within our patient cohort, those with ER-positive tumours received adjuvant endocrine therapy (mostly tamoxifen). Taking this into account, CA IX remained an independent prognostic factor.

Data regarding adjuvant chemotherapy were available for 126 of the 144 patients. Of these, 39 received adjuvant chemotherapy (mostly CMF). Of 31 patients with CA IX positivity and available data, only nine received adjuvant chemotherapy. Thus, based on these numbers, we are unable to draw conclusions regarding a predictive role for CA IX and benefit from adjuvant chemotherapy.

We certainly agree that the potential predictive value of CA IX in determining the most appropriate adjuvant therapy is interesting and reiterate our concluding remarks:

‘…CA IX expression may serve as a predictive factor to guide the selection of the most appropriate adjuvant treatment modality’ and: ‘Randomised studies with translational endpoints are required to further elucidate the prognostic and predictive value of CA IX’.

The comments by Span *et al* suggesting that CA IX-positive patients derive little or no benefit from standard treatments raise the important question whether these patients should therefore receive no treatment, or are actually candidates for targeting with potentially more effective treatment. We recognise that the utility of CA IX expression is currently experimental and the issue of both prognostic and predictive status will only be clearly resolved when investigated in a large series of patients treated within randomised trials of different therapies.
